# Coiled-Coil and C2 Domain-Containing Protein 1A (CC2D1A) Promotes Chemotherapy Resistance in Ovarian Cancer

**DOI:** 10.3389/fonc.2019.00986

**Published:** 2019-10-01

**Authors:** Sanjeev Kumar, Derek B. Oien, Ashwani Khurana, William Cliby, Lynn Hartmann, Jeremy Chien, Viji Shridhar

**Affiliations:** ^1^Department of Obstetrics and Gynecology, Mayo Clinic, Rochester, MN, United States; ^2^Division of Experimental Pathology and Laboratory Medicine, Mayo Clinic, Rochester, MN, United States; ^3^Department of Medical Oncology, Mayo Clinic, Rochester, MN, United States; ^4^Department of Biochemistry and Molecular Medicine, University of California Davis Health, Sacramento, CA, United States

**Keywords:** CC2D1A, Freud1, ovarian cancer, prognostic marker, chemotherapy resistance, treatment-refractory tumor, carboplatin, paclitaxel

## Abstract

Recurrence within 6 months of the last round of chemotherapy is clinically defined as platinum-resistant ovarian cancer. Gene expression associated with early recurrence may provide insights into platinum resistant recurrence. Prior studies identified a 14-gene model that accurately predicted early or late recurrence in 86% of patients. One of the genes identified was *CC2D1A* (encoding coiled-coil and C2 domain containing 1A), which showed higher expression in tumors from patients with early recurrence. Here, we show that CC2D1A protein expression was higher in cisplatin-resistant ovarian cancer cell lines compared to cisplatin-sensitive cell lines. In addition, immunohistochemical analysis of patient tumors on a tissue microarray (*n* = 146) showed that high levels of CC2D1A were associated with a significantly worse overall and progression-free survival (*p* = 0.0002 and *p* = 0.006, respectively). To understand the contribution of CC2D1A in chemoresistance, we generated shRNA-mediated knockdown of CC2D1A in SKOV3ip and PEO4 cell lines. Cell death and clonogenic assays of these isogenic clonal lines clearly showed that downregulation of CC2D1A resulted in increased sensitivity to cisplatin and paclitaxel in ovarian cancer cells. Moreover, nude mice bearing SKOV3ip xenografts with stably downregulated CC2D1A were more sensitive to chemotherapy as evidenced by a significantly longer survival time compared to xenografts derived from cells stably transduced with non-targeting shRNA. These results suggest CC2D1A promotes chemotherapy resistance in ovarian cancer.

## Introduction

The American Cancer Society estimates 22,500 women will be diagnosed with ovarian cancer in the United States with 14,000 deaths attributed to this disease in 2019. This has not significantly changed from recent past estimates (1) and only slightly decreased from 16,100 estimated deaths about 15 years ago (2). These statistics support the fact that amongst the common cancers of the reproductive tract in women, ovarian cancer carries the highest mortality rates. These high mortality rates of ovarian cancer are often attributed to advanced-stage diagnosis, unfavorable histologic subtypes, and disease recurrence.

The vast majority of women with epithelial ovarian cancer are diagnosed with advanced-stage disease (stage III and IV) (3). Lifelong complete remission after such a diagnosis is rare as the majority of women eventually succumb to their disease. The combination of a platinum and a taxane agent has been the standard frontline chemotherapy of ovarian cancer for over two decades (4), and the addition of recently approved bevacizumab has only modestly lengthened remission periods by months in clinical trials (5). With the utilization of frontline platinum-based chemotherapy in advanced-stage ovarian cancer patients, ~50% achieve durable remission, 20–30% have variable response whereas the remaining 20–30% may have the persistent or progressive disease (6). Unfortunately, a large fraction of the patients who initially respond to chemotherapy eventually relapse, develop resistance to chemotherapy, and ultimately die of their disease after receiving several different chemotherapy regimens.

Resistance to platinum agents is multifactorial, and several mechanisms contribute to platinum resistance (7). These molecular and cellular mechanisms can be put into four conceptual categories; namely, “pre-target” resistance, “on-target” resistance, “post-target” resistance, and “off-target” resistance (8). “Pre-target” resistance mechanisms include alterations in drug metabolism, inactivation, and efflux before platinum agents reach to the primary target, DNA. Copper transporter and MDR efflux transporter acting on glutathione-conjugated platinum have been implicated in “pre-target” resistance (9–11). In recent studies, tumor-associated stromal cells also contribute to platinum resistance through reduced glutathione regeneration, and this resistance mechanism is antagonized by tumor-infiltrated effector T cells through interferon (IFN)-γ (12). Relevant to the “on-target” resistance mechanisms are molecular mechanisms associated with DNA repair efficiency, including the restoration of homologous recombination repair function through revertant mutations as well as overexpression of DNA repair proteins, such as ERCC1 (13). Post-target resistance includes molecular mechanisms promoting anti-apoptotic signaling that counteracts the pro-apoptotic signaling induced by platinum agents (8).

Resistance to chemotherapy is a problem of monumental proportion for ovarian cancer patients (14). Discovering new drug targets for adjuvant treatment as well as the ability to predict chemotherapy resistance would potentially be a major benefit for patients. Previously, our group identified a 14-gene panel as a pre-chemotherapy predictive model of early relapse after platinum and paclitaxel treatment in ovarian cancer (15). That study examined genetic expression data of chemotherapy-naïve, advanced-stage tumor samples and applied a supervised learning algorithm to generate the early recurrence model. In additional tumor samples for testing, the 14-gene predictive model accurately (*p* < 0.05) predicted early or late recurrence in >80% of patients. Since early recurrence after primary chemotherapy is indicative of chemoresistance (16), the 14-gene panel was hypothesized to contribute to the relative ineffectiveness and resistance to platinum and paclitaxel treatment.

Although the standard-of-care chemotherapy consists of a platinum agent and a taxane agent, platinum agents are considered the most active in the combination. Accordingly, resistance to platinum agents results in treatment failure. Although taxane agents significantly improve the therapeutic effects of platinum agents, a single taxane agent is not sufficient to achieve durable response as a second-line chemotherapy (17). In this regard, it may be important to understand molecular determinants, such as TGFBI (18), that modulate paclitaxel sensitivity so that patients in platinum-resistant setting can be appropriately selected for maximal benefits with taxane-based chemotherapy in the second-line setting.

The identification of *CC2D1A* (coiled-coil and C2 domain containing 1A, identified as *FLJ20241*) as one of the 14 genes may be of particular interest regarding platinum agent efficacy (15). CC2D1A supports nuclear factor-κB (NFκB) activation (19), and inhibition of NFκB activation can increase cisplatin efficacy in ovarian cancer models (20). The objective of the present study is to investigate if *CC2D1A*, one of the constituents of the 14-gene panel predicting early recurrence in ovarian cancer, modulates chemotherapy resistance in ovarian cancer.

## Materials and Methods

### Cell Culture, Transfection, and Drug Treatment

Ovarian serous cystadenocarcinoma SKOV3 and human embryonic kidney HEK293T cell lines were obtained from the American Type Culture Collection. Ovarian cystadenocarcinoma PEO1 and PEO4 cells were obtained from Dr. Taniguchi (Fred Hutchinson Cancer Research Center, Seattle, Washington) and cultured as previously described (21). OV167 and ovarian surface epithelial cells were cultured as previously described (22, 23). A2780, C70, and C200 were obtained on an MTA from Fox Chase Cancer Center, Philadelphia, PA and cells were cultured as previously reported (24). The metastatic SKOV3ip cell line (25) was a kind gift from Dr. Ellen Vitetta (UT Southwestern Graduate School of Biomedical Science, Dallas, Texas). Cisplatin and paclitaxel (Sigma-Aldrich) were dissolved in DMSO prior to use at indicated concentrations. GI_50_ refers to the drug concentration that produces 50% inhibition of cell growth. Pharmaceutical grade carboplatin (Teva) and paclitaxel (Actavis) for injection were used in mouse studies.

### Generation of CC2D1A Polyclonal Antibodies

All animal experiments were conducted according to an approved Institutional Animal Care and Use Committee protocol. His-tagged N-terminal and C-terminal portions of CC2D1A were generated using bacterial expression system and used as antigens to generate rabbit polyclonal antibodies against the N-terminus and C-terminus of CC2D1A. Affinity purified CC2D1A antibodies were generated using corresponding antigens immobilized to AminoLink Resin (Pierce) and eluted with 100 mM glycine-HCl, pH 3.0 as previously described (26, 27). Both N-terminus and C-terminus polyclonal antibodies show specific immunoreactivity to endogenous CC2D1A in cancer cell lines.

### Transfection With shRNA

Short-hairpin RNAs (shRNAs) cloned into the lentivirus vector pLKO.1-puro were chosen from the human library (MISSION TRC-Hs 1.0) and purchased as glycerol stock from the RNA Interference Shared Resource (RISR) core at the Mayo Clinic. The non-targeting control (NTC) shRNA (Sigma) contains a hairpin insert that will generate siRNAs, but contains five base pair mismatches to any known human gene. Target sequence for Sh-1 *CC2D1A* shRNA (#3278) was: CGAACCAGACAAGCAGACAAT and for Sh-2 *CC2D1A* shRNA (#2128) was: CCACTCAAACCAATTCACCCA. Lentivirus particles were produced by transient transfection of two different plasmids targeting *CC2D1A* (pLKO.1-CC2D1A-2) and pLKO.1 NTC along with packaging vectors (pVSV-G and pGag/pol) in HEK293T cells as previously described (28).

### Western Blot Analysis and Subcellular Fractionation

Western blot was performed as previously described (29). Briefly, proteins were transferred to nitrocellulose membranes and blocked with 5% milk in tris-buffered saline with 0.5% Tween-20. Membranes were probed with CC2D1A antibody, and β-actin (Sigma) or GAPDH (Cell Signaling) antibodies as a protein loading control. Blots then probed with horseradish peroxidase-conjugated secondary antibodies (Cell Signaling). Bands were detected by chemiluminescence and visualized by autoradiography. Densitometric analysis utilized triplicate experimental data with Scion Image software. To analyze subcellular localization, SKOV3 cells lysates were separated into cytoplasmic and nuclear fractions using the Thermo Scientific NE-PER Nuclear and Cytoplasmic Extraction Reagents following the manufacturer's protocol. Equal amounts of protein from cytoplasmic and nuclear fractions were subjected to western blot analysis using CC2D1A, α-tubulin (Sigma), and PARP (Cell Signaling) antibodies.

### Cell Survival Assay

Stable *CC2D1A* knockdown clones and the respective control cell cultures were propagated. About 5,000 cells were seeded in 96-well plates and the experiments were repeated in the triplicates. To quantify viable cells, wells were incubated for 1 h with 3-(4,5-dimethylthiazol-2-yl)-2,5-diphenyltetrazolium bromide (MTT) stain by adding 2 μl of 5 mg/ml MTT into each well containing 100 μL of culture medium. Cells were then washed and solubilized with DMSO. Absorbance was read at 570 nm and survival was calculated as a percentage of controls. Proportionate cell death was also confirmed with concurrent manual cell counting.

### Clonogenic Survival Assays

Stable *CC2D1A* knockdown clones and the respective negative controls were seeded at 1,000 cells/well in 6-well plates, cultured overnight for attachment, and treated with various concentrations of cisplatin for 24 h by methods described previously (30). Subsequently, these were then incubated in drug-free medium for 2 weeks. Colonies were monitored daily and stained with Coomassie blue at the end for counting. Experiments were replicated twice in triplicates.

### Mice Studies

All animal experiments were conducted according to an approved Institutional Animal Care and Use Committee protocol. Female nude mice (age 6–7 weeks) were purchased from the National Cancer Institute. The PEO4 or SKOV3ip cultured cells were washed twice, counted, and resuspended in buffer at 2 × 10^6^/100 μL. A volume of 100 μL was injected into the peritoneal cavity of the study mice (day 0). Treatment groups contained 9 mice. Treatment with carboplatin and paclitaxel was started 14 days after inoculation. Treatment was continued at 16 mg/kg every 4 days for carboplatin and at 20 mg/kg every 4 days for paclitaxel, respectively (31). Both medications were given by the intraperitoneal route. Mice were sacrificed when obvious tumor burden hampered their movement or feeding behavior on daily inspection or at the completion of 12 weeks, whichever came first. Tumor distribution and weight were noted at the time of sacrifice.

### Tissue Microarray (TMA) Construction

Archived epithelial ovarian cancer specimens were utilized for TMA construction after human tissue use approval from the Mayo Clinic Institutional Review Board. All specimens were collected at the time of primary surgical cytoreduction before any chemotherapy exposure. The TMAs were constructed using a custom-fabricated device that utilized a 0.6-mm tissue corer and a 240-capacity recipient block. Three cores from each specimen were included. Cores of liver tissue, normal ovarian tissue, and human placenta were used as fiducial markers and controls for immunohistochemistry reactions. Five millimeter sections were cut from the TMA blocks.

### Immunohistochemistry and Digital Imaging for TMAs

Serial dilutions of CC2D1A antibody were tested on sample ovarian cancer tissue until a reproducible pattern of staining intensity emerged at 1:50 dilution. Subsequently, immunohistochemistry for CC2D1A in the ovarian cancer TMAs was performed as previously described (26). Digital imaging was performed using a BLISS “Virtual Microscopy” microscope and computer system (Bacus Laboratories Inc.). The system consists of a Zeiss Axioplan microscope with computer-interfaced electronic stage controls and a high-resolution 3CCD video camera. The Tracer program included in the BLISS system was used for automated slide scanning to create a virtual microscope slide. The CC2D1A immunohistochemistry staining pattern was classified by two independent blinded investigators (including an experienced pathologist) into high and low according to well established and validated criteria (32, 33). Non-protected health information data from patient charts were retrieved by research staff not familiar with the staining pattern of CC2D1A. Finally, the survival analysis based on high vs. low CC2D1A expression pattern was performed by an independent statistical team, blinded to the rest of the experimental analysis or patient outcomes.

### Survival Analysis in Ovarian Cancer

Key clinical variables for ovarian cancer patients included in the TMAs were extracted and stratified according to the dichotomous high/low levels of CC2D1A expression (32, 33). Because of the interest in prognosis and chemosensitivity, we performed survival analysis for the entire cohort of ovarian cancer patients as well as patients who had received chemotherapy. A 2-sided χ^2^ analysis was performed using SAS statistical software between the two groups (low vs. high CC2D1A) for the nominal or ordinal variables. Women who did not provide consent for the use of their medical information in medical research were excluded from the study per the Minnesota Statute for Use of Medical Information in Research. Optimal surgical cytoreduction was defined as residual disease (≤10 mm tumors) after surgery. Where residual disease was not indicated in the operative note, those patients were classified as unknown in terms of surgical cytoreduction.

### Statistics

Two-tailed Student's *t*-test, ANOVA followed by Newman-Keuls test, and 2-sided χ^2^ analyses were performed using Prism 3.0 (GraphPad Software) or SAS statistical software where appropriate. *p* < 0.05 and α = 0.05 were considered statistically significant.

## Results

### CC2D1A Is Expressed in Malignant and Non-malignant Ovarian Tissues

**Figure 1:**
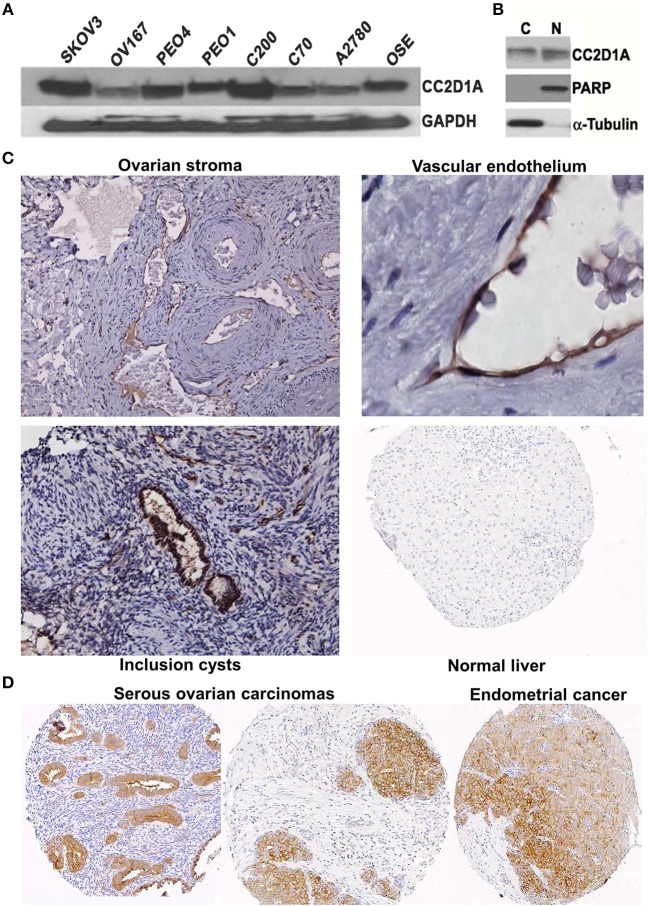
CC2D1A expression in ovarian cancer cells, tumor tissues, and non-malignant tissues. **(A)** CCD2D1A was detected in whole cell lysates from SKOV3, OV167, PEO4, PEO1, C200, C70, and A2780 ovarian cancer and ovarian surface epithelial (OSE) cells. **(B)** SKOV3 cells were separated into cytoplasmic “C” and nuclear “N” subcellular fractions and detected using CC2D1A antibody. α-tubulin, and PARP were detected as fraction controls. **(C)** Immunohistochemical staining using affinity purified CC2D1A antibodies in ovarian stroma (*upper left*), vascular endothelium (*upper right*), inclusion cysts (*lower left*), and normal liver (control; *lower right*). **(D)** CC2D1A immunohistochemical staining in tissue microarray samples of serous ovarian carcinomas (*left and middle*) and serous endometrial cancer (*right*).

We had previously reported that elevated *CC2D1A* mRNA was associated with early recurrence in ovarian tumors (15). To determine if CC2D1A protein is detectible in malignant and non-malignant ovarian tissues, we analyzed human cell lines and tissues. Western blot analysis detected the presence of CC2D1A at 100 kDa in various ovarian cancer cell lines (Figure 1A). CC2D1A is present in both the cytoplasmic as well as in the nuclear compartments of SKOV3 cells (Figure 1B), which is consistent with prior reports for CC2D1A functions in transcriptional regulation and being a scaffold protein in Akt signaling (34). For normal human ovary tissue, CC2D1A was expressed at low levels in the ovarian stroma (Figure 1C). CC2D1A was predominantly expressed in the vascular endothelium and inclusion cysts. As a control, normal liver tissue did not have detectable levels of CC2D1A expression. The expression pattern of CC2D1A in serous ovarian cancer or papillary serous endometrial cancer is also markedly higher than in surrounding stromal tissue (Figure 1D). These results suggest the CC2D1A protein is expressed in ovarian tissues, and may be expressed at higher levels in some malignant ovarian cells.

### Elevated CC2D1A Expression Is Associated With Poor Prognosis in Ovarian Cancer

**Figure 2:**
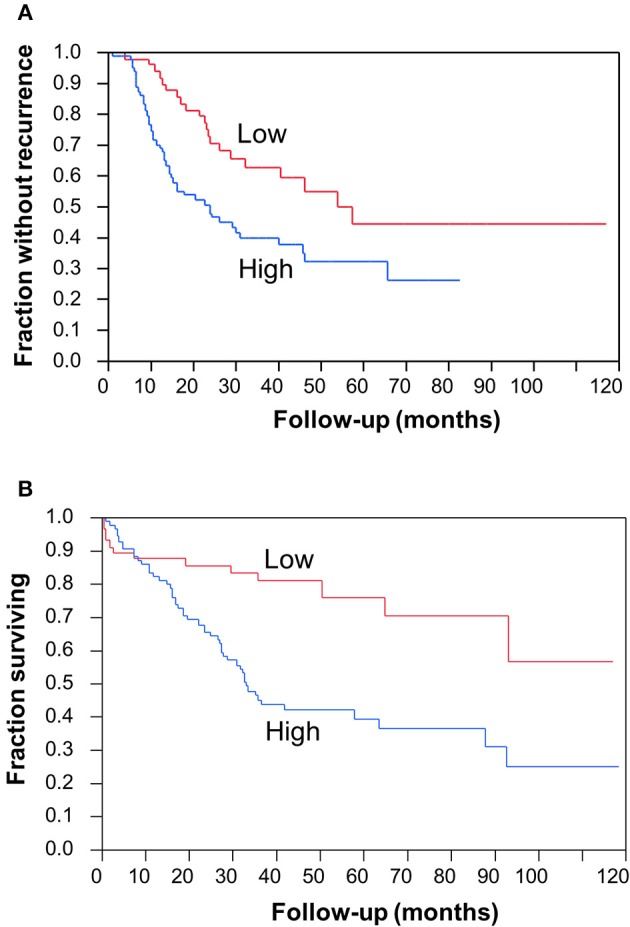
Clinical significance of CC2D1A expression in ovarian cancer. Kaplan–Meier survival analysis was performed in 146 patient samples to evaluate CC2D1A protein expression levels with progression-free patient survival. **(A)** Statistically significant (*p* = 0.006) association was found with CC2D1A protein expression and progression-free survival. Median progression-free survival for patients expressing low CC2D1A was 57 months. Median progression-free survival for patients expressing high CC2D1A tumors was 24 months. **(B)** Statistically significant (*p* = 0.0002) association was found with CC2D1A protein expression and overall survival. Median overall survival for patients expressing low CC2D1A tumors was 93 months. Median overall survival for patients expressing high CC2D1A tumors was 31 months.

To determine the clinical significance of CC2D1A expression in primary ovarian carcinomas, we analyzed CC2D1A expression in 146 carcinoma samples by immunohistochemistry (Table 1). CC2D1A expression was initially quantified by semi-quantitative scores representing absence (0), low (1), moderately high (2), and high levels (3) of CC2D1A expression. These expression scores were further dichotomized as low (score 0, 1) or high (score 2, 3) levels. CC2D1A expression was low in 40% (*n* = 59) where as it was high in 60% (*n* = 87) of samples. The majority of the tumors were high grade serous histology, diagnosed at an advanced FIGO stage, and treated by optimal surgical cytoreduction followed by platinum based chemotherapy. The clinical variables were not different between the groups of tumors with high CC2D1A expression compared to those with low expression. The median progression-free survival for the entire group was 31 months. The median progression-free survival for tumors with low CC2D1A expression was 57 months, which was significantly different (*p* = 0.006) compared to 24 months for those with high expression (Figure 2A). Similarly, the overall survival for the patients with low CC2D1A expression was significantly better (*p* = 0.0002) than those with high expression (Figure 2B; Table 1). In a subgroup analysis focusing on advanced-stage (stages III and IV) serous epithelial ovarian cancer (*n* = 93), the median survival for patients with low CC2D1A expression was 93 months as compared to 31 months for those with high expression (log-rank test; *p* = 0.0003). More importantly, in additional subgroup analysis, patients were stratified based on utilization of chemotherapy (yes/no). In this analysis, the survival advantage associated with low CC2D1A expression persisted in the subgroup of patients receiving chemotherapy (*p* = 0.0001), but there was not a difference for the subgroup of patients not receiving chemotherapy (*p* = 0.8). These data suggest high CCD1A expression is correlated with poor progression-free and overall survival outcomes.

**Table 1:** Distribution of key variables in epithelial ovarian cancer subjects with respect to CC2D1A expression levels.

**Variable**	**Description**	**CC2D1A low (column %)**	**CC2D1A high (column %)**	***p*-value**
Total		59 (100)	87 (100)	
Age (years)	Mean	60 (*SD* = 12)	59 (*SD* = 12)	
Body mass index	Mean	32 (*SD* = 23)	31 (*SD* = 23)	
FIGO stage	I	9 (15)	12 (14)	0.8
	II	8 (14)	3 (4)	
	III	33 (56)	49 (56)	
	IV	9 (15)	23 (26)	
Grade	Low	12 (21)	11 (14)	0.4
	High	45 (79)	69 (86)	
Histology	Serous	40 (68)	53 (61)	0.4
	Non-serous	19 (32)	34 (39)	
Optimal surgical cytoreduction	Yes	42 (71)	59 (68)	0.08
	No	1 (2)	10 (12)	
	Unknown	16 (27)	18 (21)	
Frontline chemotherapy	Carboplatin + paclitaxel	48 (81)	69 (79)	0.4
	Non-platinum	0	3 (3)	
	None/unknown	11 (19)	15 (17)	
Status	Alive at time of study	46 (78)	36 (41)	<0.001
	Deceased at time of study	13 (22)	51 (59)	
5-year survival	Survivors	76%	40%	0.002

### Knockdown of CC2D1A Leads to Increased Chemosensitivity *in vitro*

**Figure 3:**
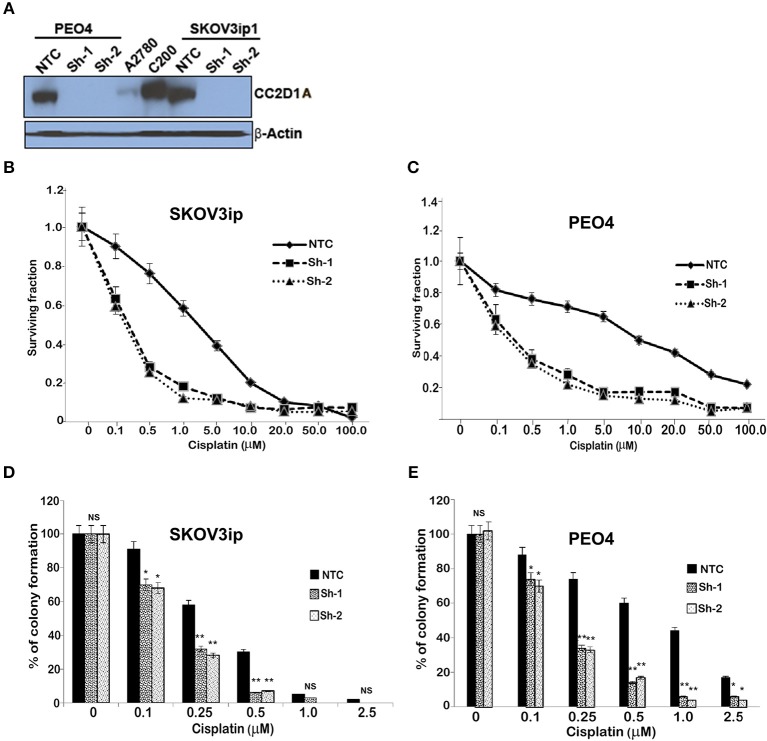
Knockdown of CC2D1A enhances cisplatin. **(A)** PEO4 and SKOV3ip1 ovarian cancer cells were grown and transduced with NTC and two different shRNA against CC2D1A (Sh-1 and Sh-2). Batch clones were generated under puromycin selection. Cells were harvested and subjected to immunoblot analysis using anti-CC2D1A and anti-β actin antibodies. **(B)** SKOV3ip1 and **(C)** PEO4 NTC, Sh-1, and Sh-2 cells were plated in 96-well plates in replicates of six and treated with indicated concentrations of cisplatin for 48 h. Cell viability was determined by MTT assay. The data represents two independent assays done in replicates of six. **(D)** SKOV3ip and **(E)** PEO4 cells plated in six-well plates were treated with indicated concentrations of cisplatin for 24 h followed by 2 weeks in drug-free medium. Colonies were stained with Coomassie blue. Experiments were replicated twice in triplicate. **p < 0.001, *p < 0.05, NS, not significant compared to NTC cells.

**Figure 4:**
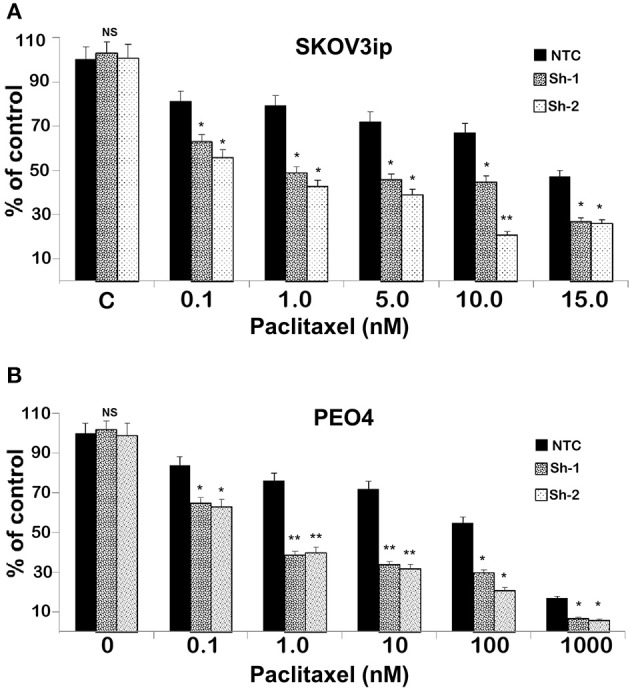
Knockdown of CC2D1A enhances paclitaxel. **(A)** SKOV3ip and **(B)** PEO4 NTC, Sh-1 and Sh-2 cells plated in 6-well plates were treated with indicated concentrations of paclitaxel for 48 h. Subsequently, the cells were washed incubated in drug-free medium for 2 weeks. Colonies were daily monitored and stained with Coomassie blue at the end of 2 weeks and counted. Experiments were replicated twice in triplicates. **p < 0.001, *p < 0.05, NS: not significant compared to NTC cells.

To test the extent to which CC2D1A expression contributes to chemotherapy resistance *in vitro*, we knocked down *CC2D1A* expression in two different ovarian cancer cell lines, PE04 and SKOV3ip (Figure 3A). Cells stably transfected with NTC showed robust expression of CC2D1A in PE04 and SKOV3ip cells. Cells stably transfected with Sh-1 or Sh-2 CC2D1A shRNA showed efficient downregulation of CC2D1A expression. We then used MTT assays to determine the sensitivity of these clones to cisplatin. The GI_50_ values of SKOV3ip cells for cisplatin in cells stably transfected NTC, Sh-1, and Sh-2 cells were 5.0, 1.2, and 0.3 μM, respectively (Figure 3B) and in PEO4 cells was 10.1 μM for NTC compared to 0.25 μM in both Sh-1 and Sh-2 (Figure 3C). Thus, knockdown of CC2D1A resulted in a dramatic reduction in the GI_50_ values in these cells. Consistent with the MTT assay, our clonogenic assays showed similar reductions of clonogenic survival in response to cisplatin for the CC2D1A-deficient Sh-1 and Sh-2 clones when compared to the NTC-transfected cells with intact CC2D1A (Figures 3D,E). Clonogenic assays in the SKOV3ip showed a decrease in paclitaxel GI_50_ from 15 nM for NTC compared to 1 nM for Sh-1 and Sh-2 (Figure 4A), and for PEO4 cells from 100 nM in NTC to <1 nM in Sh1 and Sh2 (Figure 4B). Paclitaxel insensitivity for PEO4 cells compared to A2780 cells has been previously reported (attributed to high miR-433 that was not evaluated in this study), which is consistent with the higher GI_50_ of these results and low CC2D1A expression in A2780 (Figure 3) (35). These results suggest CC2D1A reduces the efficacy of chemotherapy in ovarian cancer cells.

### Knockdown of CC2D1A Leads to Increased Chemosensitivity *in vivo*

**Figure 5:**
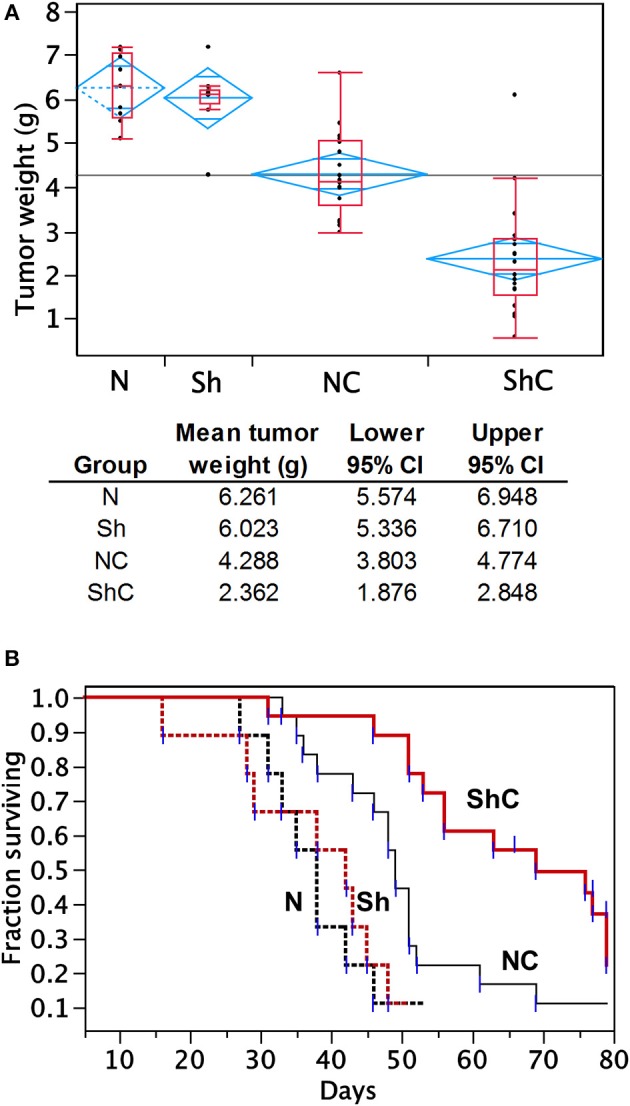
Carboplatin and paclitaxel combination treatment is more effective in PEO4 mouse xenografts with CC2D1A knockdown. **(A)** Analysis of tumor weight from mouse xenografts treated with or without chemotherapy. No significant differences in tumor weight were observed between mice bearing CCD21A-expressing (N) tumor xenograft and those bearing CCD21A-knocked down (Sh) tumor xenografts when chemotherapy was not given. Significant reduction in tumor weight was observed between mice bearing CC2D1A-expressing (NC) tumor xenograft and those bearing CC2D1A-knockdown (ShC) tumor xenografts when chemotherapy was given. **(B)** Chemotherapy significantly extends the survival of mice bearing tumor xenografts with CC2D1A knockdown. **(A)** Surviving fraction of four groups of mice with tumor xenografts (NTC-xenografts, N; CC2D1A shRNA-xenografts, Sh; NTC-xenografts treated with chemotherapy, NC; Sh-xenografts treated with chemotherapy, ShC). **(B)** Survival analysis of NTC-xenografts treated with chemotherapy (NC) vs. Sh-xenografts treated with chemotherapy, ShC). Median survival in days is shown for each group.

To determine the effect of CC2D1A expression on chemotherapy sensitivity *in vivo*, we generated tumor xenografts for the PEO4 cell line stably expressing NTC or Sh-1. These mice were then analyzed for tumor weight and survival in groups treated with and without chemotherapy, which was carboplatin and paclitaxel (Figure 5). In this study, N denotes *NTC* (CC2D1A-positive) control, *Sh* denotes stable cells transduced with CC2D1A Sh-1, and *C* denotes chemotherapy. Figure 5A represents the tumor weight distribution in various groups of mice at the time of necropsy. There was no difference in the tumor weights between NTC and CC2D1A shRNA-derived xenografts when no chemotherapy was used. In the treated group, the mean tumor weight was reduced to ~70% of the non-treatment controls for the NTC shRNA (NC) group. Further, the CC2D1A shRNA-derived xenografts (ShC) showed a marked reduction in tumor weight with chemotherapy of ~30% when compared to non-treatment groups (N & Sh) and to ~50% when compared to CC2D1A expressing xenografts with chemotherapy (NC), respectively (*p* < 0.0001). The survival of mice with CC2D1A-expressing (N) and CC2D1A-knockdown (Sh) xenografts remained similar to each other when chemotherapy was not given (Figure 5B). In contrast, introduction of chemotherapy improved survival for CC2D1A-knockdown (ShC) group to a larger extent (*p* < 0.001), and for the CC2D1A-expressing (NC) group for a smaller extent (Figure 5B). In a subgroup analysis comparing the ShC group to the NC group, the median survival of the former was 20 days longer than that of latter (Figure 5B, *p* = 0.002). Overall, these results suggest CC2D1A may not be important for ovarian cancer progression, but may be negatively associated with the tumor reduction ability of carboplatin and paclitaxel.

## Discussion

The coiled-coil and C2 domain-containing protein 1A is a highly conserved protein encoded by the *CC2D1A* gene, which was initially identified as Freud1 and a repressor of serotonin receptor 1A expression (36, 37). CC2D1A was later reported to act as a scaffold to anchor the EGFR-mediated activation of the PDK1/Akt pathway (34). Consistent with previously reported nuclear and cytoplasmic role of CC2D1A, we observed both nuclear and cytoplasmic localization for CC2D1A in cancer cells (Figure 1B).

Subsequent to the discovery of the *CC2D1A* as one of the constituent members of the 14-gene panel predicting early recurrence in ovarian cancer (15), we hypothesized that CC2D1A has a role in modulating chemoresistance of ovarian cancer. In the present study, we show (1) patients with high CC2D1A expression in their tumor samples had shorter overall and progression free survival (Figure 2), (2) cancer cells become more sensitive to both paclitaxel and cisplatin upon stable knockdown of CC2D1A (Figures 3, 4), and (3) mice bearing CC2D1A-deficient xenografts survived longer only when treated with chemotherapeutic agents (Figure 5). These data support our notion that CC2D1A expression modulates the tumor response to chemotherapy both *in vitro* and *in vivo*.

Previous reports show that the knockdown of CC2D1A sensitizes cells to etoposide or camptothecin (34). Consistent with this report, we observed that *CC2D1A* shRNA-downregulated clones in PEO4 cells were more sensitive to both cisplatin and paclitaxel both *in vitro* and *in vivo*. The molecular pathway used by SKOV3ip cells with CC2D1A knockdown to sensitize these cells to cisplatin and paclitaxel mediated cytotoxicity is currently unknown. CC2D1A is a potent activator of NFκB (27). In ovarian cancer, activation of NFκB is associated with an aggressive phenotype resulting in reduced progression-free survival (38) with resistance to cisplatin- and paclitaxel-based therapies (20, 39). Whether downregulation of CC2D1A in SKOV3ip cells attenuates NFκB-mediated chemotherapy resistance is currently under investigation.

We observed a striking difference in progression-free and overall survival between the ovarian cancers with high vs. low expression of the CC2D1A (Table 1; Figures 2A,B). We compared this to the TCGA database where *CC2D1A* amplification is more common in ovarian cancer vs. the other 31 PanCancer TCGA datasets (Supplementary Figure 1). Our data are consistent with the poor overall survival of patients with high *CC2D1A* expression in the TCGA database (Supplementary Figure 2). In the TCGA cohort, 113 of 571 cases showed alteration of *CC2D1A*. The median survival of patients with *CC2D1A* alterations was 36.62 months compared to 47.60 months in patients without *CC2D1A* alteration. This difference was statistically significant (*p* = 0.0276). Results from ovarian cancer TCGA dataset were further corroborated by immunohistochemical analysis of CC2D1A protein expression in tumor TMAs, which showed significantly higher 5-year survival rate in patients with low CC2D1A expression than in patients with high CC2D1A expression in tumor samples (Figure 2). Table 1 demonstrates that the distribution of the key characteristics such as age, stage, grade, histology, surgical effort, and chemotherapy was statistically similar between the two groups in the TMA study. Hence, the association of CC2D1A-rich tumors with that of poorer survival seems to point toward chemotherapy resistance and earlier recurrence rather than toward any other confounding variable. A closer analysis of our mice survival data demonstrates that the Kaplan–Meier curves of mice bearing the xenografts derived from the NTC-transfected cells and the corresponding CC2D1A deficient clone overlapped. Treatment with chemotherapy yielded better survival in both the groups, respectively. Therefore, we surmised that in the xenograft model the CC2D1A knockdown did not affect tumorigenicity but exhibited marked effects on chemotherapy sensitivity. To the best of our knowledge, this is the first report of a gene affecting chemotherapy resistance without affecting tumorigenicity.

Although most ovarian cancer cases are initially sensitive to current chemotherapies, the emergence of the chemotherapy-resistant tumors leads to ~60% of the women dying from the disease (40). This means that the overall cure rates have not improved. Only 20–25% of women diagnosed with advanced stage disease are alive at 5 years (41). One of the reasons for this dismal statistics is that the minimal residual disease, present after optimal surgery and chemotherapy, is resistant to therapy and emerges as a clinically unmanageable disease. In this context, the study by Tewari et al. that compared a large set tumors obtained from primary ovarian tissues vs. metastatic sites for *in vitro* drug response demonstrated *in vitro* drug resistance at recurrence was not influenced by intervening therapy (42). Consistent with this data, another report with non-randomized study suggests that patients who receive agents to which their tumor is resistant to *in vitro* chemotherapy progress more rapidly and have shorter disease-free survival times (43–45). All of these studies support the theory that the molecular alterations promoting drug resistance may occur early in the carcinogenic process and persist in the metastatic sites. Therefore, the identification of genetic changes in the tumor at the time of diagnosis after the debulking surgery may help clinicians in guiding a targeted therapy. We believe the identification of CC2D1A as a marker of early recurrence in pretreatment specimens will be clinically beneficial to identify patients who may not respond to conventional chemotherapy and serve as a guide for choosing a more aggressive therapy. Finally, further studies are needed to validate our findings on a larger scale and to define the biological role of CC2D1A in health and disease.

In this aspect, the potential role of CC2D1A in NFκB signaling warrants further investigation. NFκB signaling has been implicated in cisplatin resistance through on-target and post-target mechanisms involving the transcriptional regulation of DNA repair genes and anti-apoptotic survival signaling (46). In addition, NFκB-mediated histone modifications contribute to cisplatin resistance (47), and therefore, the extent to which CC2D1A affects NFκB-mediated histone modifications should be investigated. Inhibiting NFκB signaling via regulation of the TGFß pathway has also been shown to promote paclitaxel sensitivity in ovarian cancer cells (48). TGFß modulation of paclitaxel sensitivity in ovarian cancer has been previously established (18), and the extent to which CC2D1A affects NFκB signaling similar to TGFß pathways should also be investigated.

In summary, our findings reveal that CC2D1A can be used as a marker of poor survival or chemotherapy resistance in ovarian cancer. Predicting the response to treatment outcomes based on CC2D1A expression may have clinical benefit in choose the best therapeutic approach.

## Data Availability Statement

All datasets generated for this study are included in the manuscript/Supplementary Files.

## Ethics Statement

The studies involving human participants were reviewed and approved by Mayo Clinic Institutional Review Board. The patients/participants provided their written informed consent to participate in this study. The animal study was reviewed and approved by Mayo Clinic Institutional Animal Care and Use Committee.

## Author Contributions

SK, JC, and VS were involved in the initial development of this project. Data were generated and analyzed by SK, DO, AK, WC, LH, and JC. SK and DO prepared this manuscript. All authors were involved in the editing of this manuscript.

### Conflict of Interest

The authors declare that the research was conducted in the absence of any commercial or financial relationships that could be construed as a potential conflict of interest.
