# Supplementary material for: Coiled-Coil and C2 Domain-Containing Protein 1A (CC2D1A) Promotes Chemotherapy Resistance in Ovarian Cancer
Source: Front Oncol. 2019 Oct 1;9:986. doi: 10.3389/fonc.2019.00986 (PMC6779793; doi:10.3389/fonc.2019.00986)
Supplement: Supplementary file 1 [file Data_Sheet_1.pdf]

Supplementary Material

Coiled-coil and C2 domain-containing protein 1A (CC2D1A) promotes chemotherapy resistance in ovarian cancer

Sanjeev Kumar[1], Derek B. Oien[2], Ashwani Khurana[2], William Cliby[1], Lynn Hartmann[3], Jeremy Chien[4], Viji Shridhar[2]*

* **Correspondence:** Viji Shridhar

1 Supplementary Figures

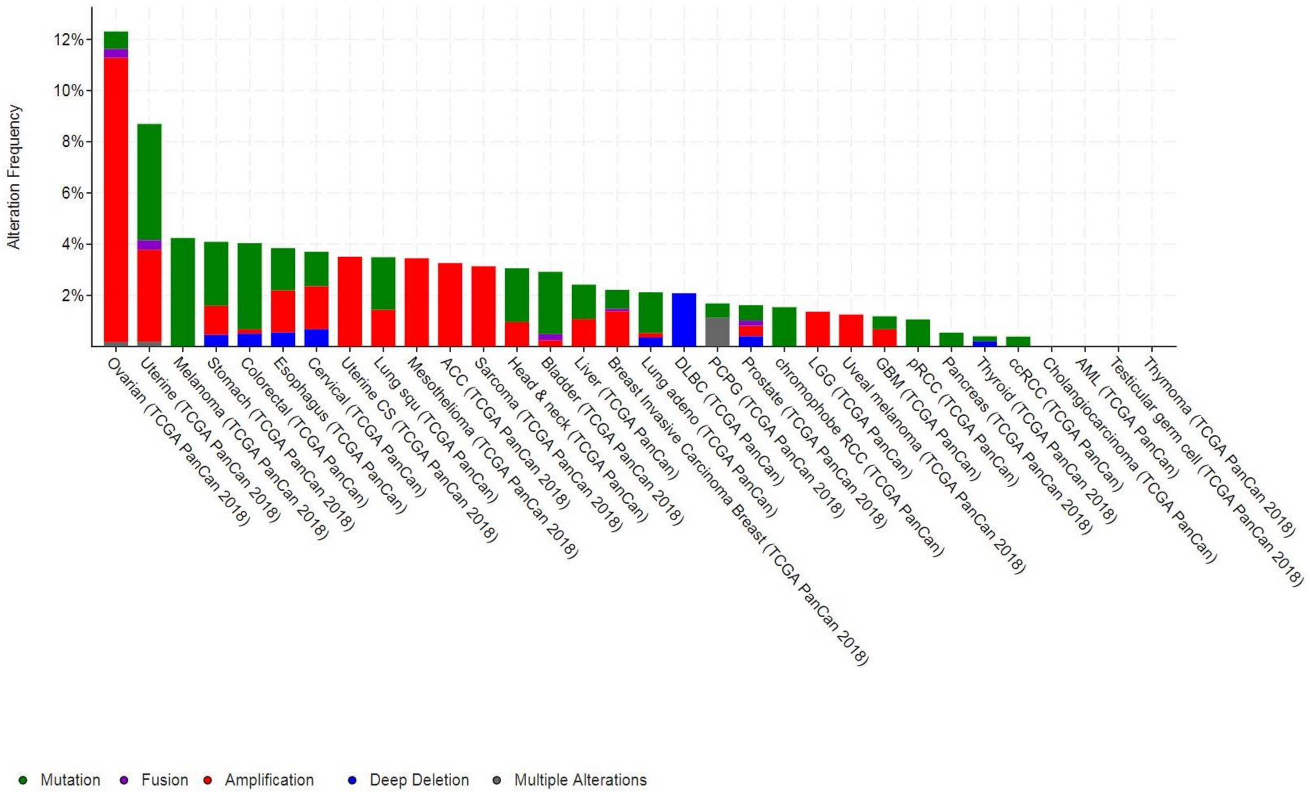

Supplementary Figure 1. Genetic alteration frequency of CC2D1A for the 32 TCGA PanCaner datasets. Ovarian cancer has the highest CC2D1A alteration frequency (>12% samples), with the majority of alterations being gene amplifications. Graph generated from cBioPortal based on data from the TCGA Research Network (Cerami et al., 2012; Gao et al., 2013).

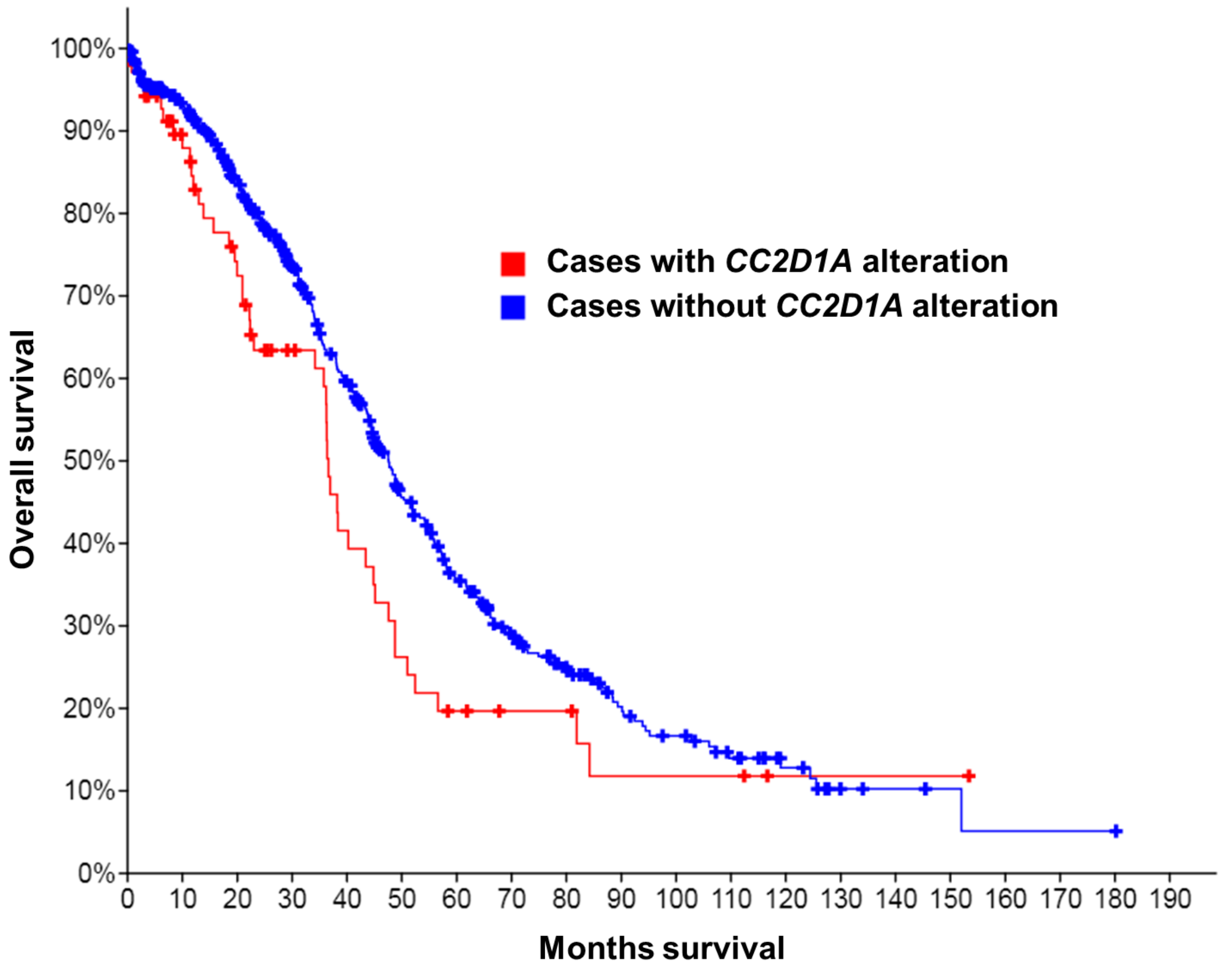

Supplementary Figure 2. Kaplan-Meier survival analysis of altered expression for CC2D1A in ovarian cancer TCGA PanCancer cohort with 571 cases (Hoadley et al., 2018). The median survival of patients with CC2D1A alterations was 36.62 months compared to 47.60 in patients without CC2D1A alteration (log-rank test $p = 0.0276$). Graph generated from cBioPortal based on data from the TCGA Research Network (Cerami et al., 2012; Gao et al., 2013).

Supplementary References

Cerami, E., Gao, J., Dogrusoz, U., Gross, B.E., Sumer, S.O., Aksoy, B.A., et al. (2012). The cBio cancer genomics portal: an open platform for exploring multidimensional cancer genomics data. *Cancer Discov* 2(5)**,** 401-404. doi: 10.1158/2159-8290.cd-12-0095.

Gao, J., Aksoy, B.A., Dogrusoz, U., Dresdner, G., Gross, B., Sumer, S.O., et al. (2013). Integrative analysis of complex cancer genomics and clinical profiles using the cBioPortal. *Sci Signal* 6(269)**,** pl1. doi: 10.1126/scisignal.2004088.

Hoadley, K.A., Yau, C., Hinoue, T., Wolf, D.M., Lazar, A.J., Drill, E., et al. (2018). Cell-of-Origin Patterns Dominate the Molecular Classification of 10,000 Tumors from 33 Types of Cancer. *Cell* 173(2)**,** 291-304.e296. doi: 10.1016/j.cell.2018.03.022.
